# Tryptophan Metabolites Regulate Neuropentraxin 1 Expression in Endothelial Cells

**DOI:** 10.3390/ijms23042369

**Published:** 2022-02-21

**Authors:** Romain Vial, Stéphane Poitevin, Nathalie McKay, Stéphane Burtey, Claire Cerini

**Affiliations:** 1 Centre de Néphrologie et Transplantation Rénale, Assistance Publique-Hôpitaux de Marseille, Hôpital de La Conception, 147 Boulevard Baille, 13005 Marseille, France; romain.vial@ap-hm.fr (R.V.); stephane.burtey@univ-amu.fr (S.B.); 2 Centre de Recherche en Cardiovasculaire et Nutrition (C2VN), Institut de la Santé et de la Recherche Médicale (INSERM), Institut National de la Recherche pour l’Agriculture, l’Alimentation et l’Environnement (INRAE), Aix Marseille University, 13005 Marseille, France; stephane.poitevin@univ-amu.fr (S.P.); nathalie.mc-kay.1@univ-amu.fr (N.M.)

**Keywords:** aryl hydrocarbon receptor, indolic uremic toxins, indoxyl sulfate, indole-3-acetic acid, neuropentraxin 1

## Abstract

In patients with chronic kidney disease (CKD) and in animal models of CKD, the transcription factor Aryl Hydrocabon Receptor (AhR) is overactivated. In addition to the canonical AhR targets constituting the AhR signature, numerous other genes are regulated by this factor. We identified neuronal pentraxin 1 (NPTX1) as a new AhR target. Belonging to the inflammatory protein family, NPTX1 seems of prime interest regarding the inflammatory state observed in CKD. Endothelial cells were exposed to tryptophan-derived toxins, indoxyl sulfate (IS) and indole-3-acetic acid (IAA). The adenine mouse model of CKD was used to analyze NPTX1 expression in the burden of uremia. NPTX1 expression was quantified by RT-PCR and western blot. AhR involvement was analyzed using silencing RNA. We found that IS and IAA upregulated NPTX1 expression in an AhR-dependent way. Furthermore, this effect was not restricted to uremic indolic toxins since the dioxin 2,3,7,8-Tetrachlorodibenzo-p-dioxin (TCDD) and the tryptophan photoproduct 6-formylindolo[3,2-b]carbazole (FICZ) do the same. In CKD mice, NPTX1 expression was increased in the aorta. Therefore, NPTX1 is a new target of AhR and further work is necessary to elucidate its exact role during CKD.

## 1. Introduction

Chronic kidney disease (CKD), affecting 11% of the world’s population, arises from diseases that occur at a variable pace, leading to progressive and irreversible destruction of the kidney [1]. The continuous loss of nephrons during CKD leads to perturbations of the internal environment, which worsen during the progression of the disease. Numerous solutes, called uremic toxins, accumulate in the blood and tissues of patients during CKD [2]. Accumulation of uremic toxins impacts all functions of the body and patients with CKD display numerous health problems, such as anemia, increased bone fragility and cancer risk, cognitive impairment, and gastrointestinal disturbances [1]. However, the most preoccupant problem remains the high rate of cardiovascular disease (CVD), which cannot be explained by the presence of classical cardiovascular risk factors [3,4]. Endothelium dysfunction [5,6,7,8,9], chronic inflammation [10,11,12], procoagulant state [10,11,12,13,14,15,16] and uremic toxins [2,8,15,16,17,18] are the new players of CVD associated with CKD.

Uremic toxins are chemically diverse compounds that cannot be cleared by the unhealthy kidney and their concentrations increase during the progression of CKD. Among them, those arising from tryptophan metabolism are of critical interest because of the links they display to multiple diseases [14,19]. Tryptophan is an essential amino acid; its metabolism originates from dietary proteins [14]. Once released from proteins by microbiota and host proteases, tryptophan can be either used for protein synthesis or enter the kynurenine, serotonin or indolic pathway [14]. More recently, another route for tryptophan transformation has been described, especially in the skin where UV and light can crosslink tryptophans to give the 6-formylindolo [3,2-b]carbazole (FICZ) photoproduct, which participates in circadian clock regulation [20]. Among the metabolites produced by the pathways quoted above, some of them are considered uremic toxins. This is the case for indoxyl sulfate (IS) and indole-3-acetic acid (IAA), which are especially deleterious and belong to the indolic pathway [2,21,22,23,24].

Because of the major role of the endothelium in the integrity of the cardiovascular system and to improve the knowledge of how indolic uremic toxins act on endothelial cells, we performed microarray experiments using human umbilical vein primary endothelial cells (HUVEC) exposed to IS for 4 and 24 h [24]. Among genes whose expression is upregulated by IS, we found genes involved in inflammation and thrombosis, such as *F3* (tissue factor) and *PTSG* (cyclooxygenase 2), providing in this way a mechanism that could contribute to the high cardiovascular mortality during CKD [14,24]. The gene coding for neuronal pentraxin 1 (NPTX1) was also identified as a target of IS [24]. It belongs to the pentraxin family, which also contains inflammatory proteins [25]. In addition, we showed that the increased expression of these genes depends on the activation by IS of the transcription factor Aryl Hydrocarbon Receptor (AhR). 

The aim of this work is to characterize the altered pattern of NPTX1 expression induced by IS and to confirm the central role of AhR. In addition, because the AhR response in terms of gene expression pattern exhibits agonist specificity, we have extended this work to other AhR agonists, which are: the other indolic harmful toxin, IAA; the trytophan photoproduct, FICZ; and the historical environmental agonist, the 2,3,7,8-Tetrachlorodibenzo-p-dioxin (TCDD).

## 2. Results

### 2.1. Time Effect of Indolic Uremic Toxins on NPTX1 Expression

**Figure 1:**
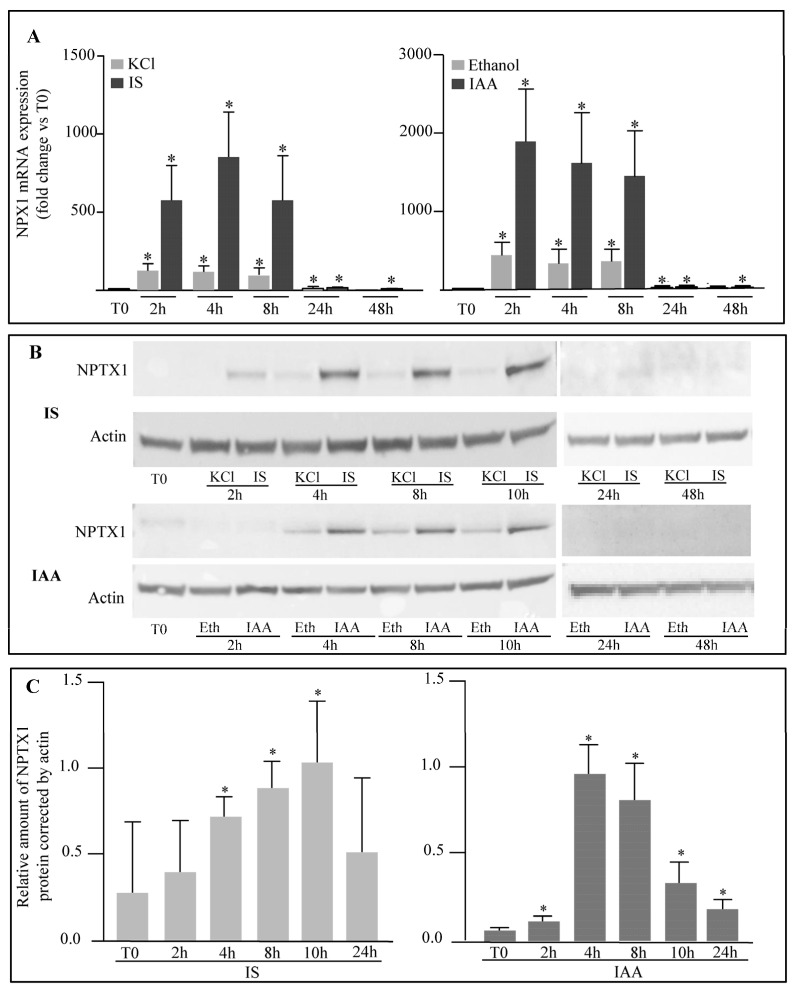
Effects of IS and IAA on NPTX1 expression. Cells were incubated with IS, IAA or with their respective controls at the same concentration [IS or KCl, 200 µM; IAA or ethanol (Eth), 50 µM] for the indicated time. (**A**) NPTX1 mRNA levels were quantified by RT-PCR (*n* = 5). (**B**) Western blots were performed with 40 µg total protein and revealed with antibodies against NPTX1 or against actin (*n* = 5). (**C**) NPTX1 protein amounts were quantified by *ImageJ* software. Wilcoxon matched-pairs signed rank test was used to compare toxins to their controls, and a *p* value < 0.05 was considered significant (*).

The uremic indolic solutes IS and IAA increased the NPTX1 mRNA level in HUVEC (Figure 1). In resting cells, just before the addition of fresh medium containing uremic toxins or their controls (T0), the amount of NPTX1 mRNA and protein was very low, nearly undetectable. After two hours of incubation, a high increase in mRNA levels could be observed in the presence of the two toxins until 8 h (Figure 1A). Then, the mRNA amount declined to reach low levels after 24 h of incubation, similar to those observed in resting cells. In the presence of IS, NPTX1 protein expression followed those of mRNA, with the highest levels reached at 10 h and a progressive return to low levels until 24 h (Figure 1B,C). The increase in mRNA levels remained significant at 24 and 48 h, although they were falling within the limit of detection. This could be due rather to the fact that mRNA levels continue to be slightly higher compared to controls than a true sustain increase. The effect of IAA on NPTX1 expression presents a pattern similar to IS except that the highest values for mRNA and protein levels were reached earlier: 2 h vs. 4 h for the mRNA and 4 h vs. 8–10 h for the protein (Figure 1B,C). 

The medium controls also induced NPTX1 expression but to a lesser extent. We have chosen to express NPTX1 mRNA levels in fold change compared to T0 to exhibit the effect of controls.

### 2.2. Dose Effect of Indolic Uremic Toxins on NPTX1 mRNA Levels

**Figure 2:**
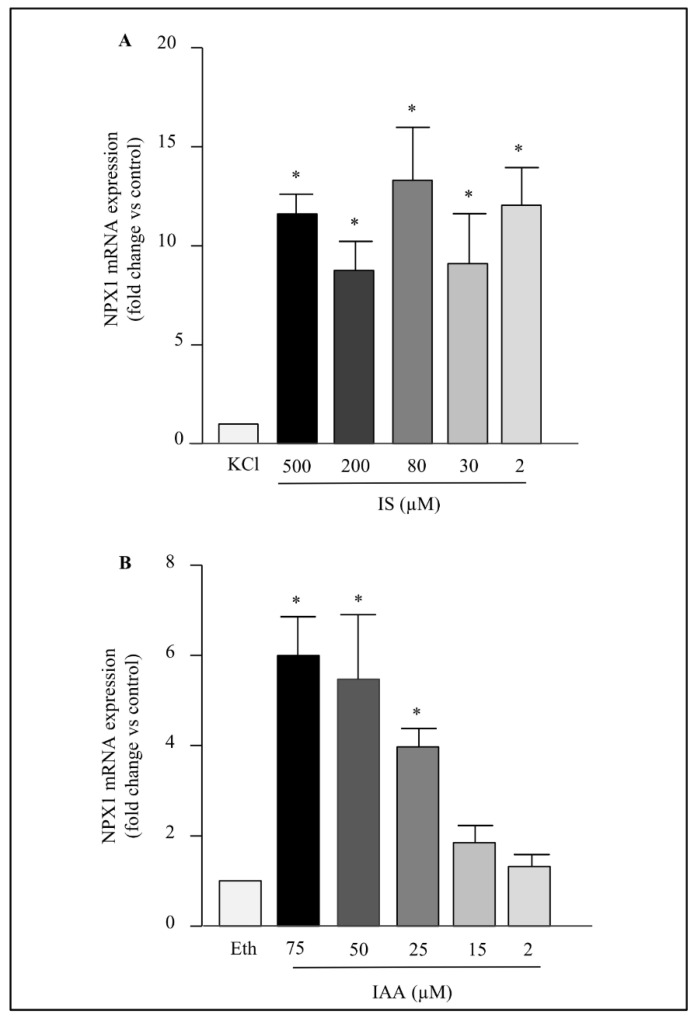
Effects of IS (**A**) and IAA (**B**) concentrations on NPTX1 mRNA levels. Cells were incubated with IS, IAA or their respective controls (KCl for IS and ethanol (Eth) for IAA) at the indicated concentrations during 4h. NPTX1 mRNA levels were quantified by RT-PCR (*n* = 5). Values are expressed as mean ± SD. Wilcoxon matched-pairs signed rank test was used and a *p* value < 0.05 was considered significant (*).

The range concentration used for the two toxins includes concentrations found in the blood of patients with CKD [2,21]. Between 500 and 2 µM, IS displayed a similar effect on NPTX1 mRNA levels compared to cells exposed to KCl (Figure 2A). In contrast, IAA showed a dose effect between 75 and 25 µM (Figure 2B). 

### 2.3. Role of Aryl Hydrocarbon Receptors in the Induction of NPTX1 Expression by Indolic Uremic Toxins

**Figure 3:**
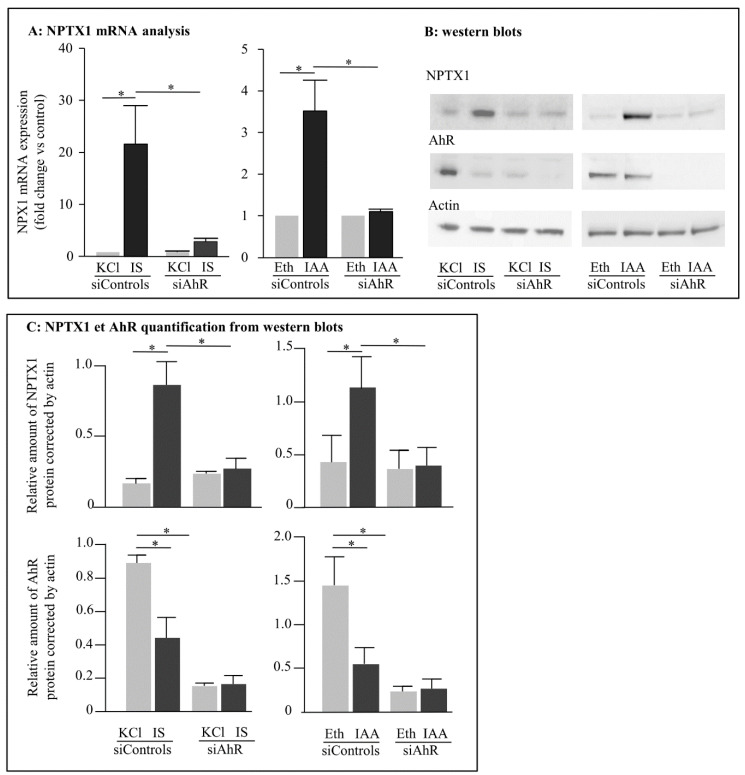
Role of AhR in the induction of NPTX1 by IS and IAA. HUVEC were transfected by si RNA controls (siControls) or directed against AhR (siAhR). 48H after transfection, HUVEC were incubated during 4h with IS (200 µM), IAA (50 µM), KCl or ethanol. (**A**) NPTX1 mRNA levels were quantified by RT-PCR (*n* = 4). (**B**) 40 µg of total protein were loaded on SDS PAGE and revealed using antibodies against AhR, NPTX1 and actin (*n* = 5). (**C**) NPTX1 and AhR protein amounts were quantified by the ImageJ software. For (**A**,**C**), values are expressed as mean ± SD. Wilcoxon matched-pairs signed rank test was used and a *p* value < 0.05 was considered significant (*).

In the presence of siRNA controls, IS and IAA increased mRNA levels of NPTX (Figure 3A). IS induced a 5-fold increase and IAA a 2.6-fold increase in NPTX1 protein level (0.880 vs. 0.177 for IS; 1.15 vs. 0.44 for IAA) (Figure 3B,C). We have previously shown that treatment of HUVEC with three siRNAs against AhR dramatically decreases mRNA and protein amounts [24] and here the percentage of reduction of AhR protein is 82% (Figure 3B). IS and IAA could no longer induce NPTX1 expression at mRNA and protein levels when AhR expression was nearly abolished by silencing RNA (Figure 3A–C). 

In addition, after activation by its ligands and translocation into the nucleus, AhR is degraded by the proteasome [24,26]. This is the reason why we can observe a decrease in AhR protein amount in the presence of IS or IAA in cells treated with siRNA controls. 

**Figure 4:**
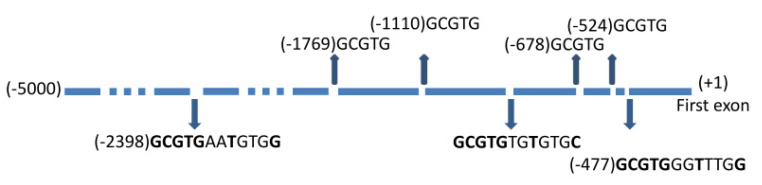
XRE sequences in the Promotor of the NPTX1gene. The first 5000 bases upstream of the first exon were presented. The three long canonical XRE sequences (5′GCGTGNNA/TNNNC/G3′) of AhR binding and the four short (5′GCGTG3′) are displaying with their respective position from the first exon.

Concordant with these results, we found 3 canonical consensus sequences for AhR binding in the promoter of the *NPTX1* gene (the blast alignment was performed on the first 5000 nucleotides localized downstream to the first exon) (Figure 4). In this canonical sequence (5′-GCGTGNNA/TNNNC/G-3′) named Xenobiotic Response Element (XRE), AhR binding to DNA required the invariant core (5′-GCGTG-3′) and the supplementary nucleotides in 3′ seem to be less indispensable [27]. For this reason, we have also noticed the four shorter core motifs found in the promoter (Figure 4). 

### 2.4. The Induction of NPTX1 Expression by AhR Activation Was Not Restricted to Indolic Uremic Solutes in Endothelial Cells

**Figure 5:**
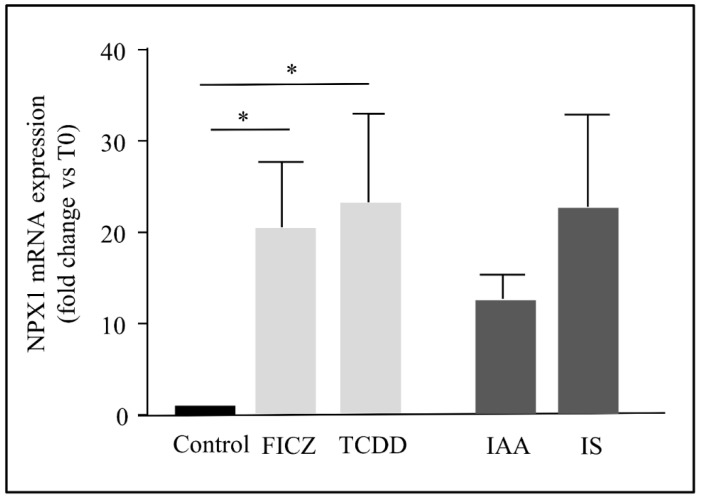
Effects of well known agonists of AhR on NPTX1 mRNA levels. Cells were incubated during 4 hours with IS (200 µM), IAA (50 µM), FICZ (10 nM), and TCDD (30 nM), or their respective controls at the same concentrations. Culture medium did not contain growth factor nor serum but 0.5% human albumin. NPTX1 mRNA levels were quantified by RT-PCR (*n* = 5). Values are expressed as mean ± SD. Wilcoxon matched-pairs signed rank test was used and a *p* value < 0.05 was considered significant (*).

To test different kinds of agonists, we used other well-known AhR agonists, 2, 3, 7, 8-tetrachlorodibenzo-p-dioxin (TCDD) and FICZ. TCDD is a well-known xenobiotic agonist and FICZ is a photoproduct of tryptophan produced in the skin. Both TCDD and FICZ could also induce to the same extent compared to indolic toxin mRNA expression of NPTX1 (Figure 5). 

In addition, the effect was also not limited to HUVEC since these toxins can also induce mRNA expression in human primary endothelial cells from coronaries (data not shown).

### 2.5. NPTX1 Expression in the Adenine Mice Model of CKD

**Figure 6:**
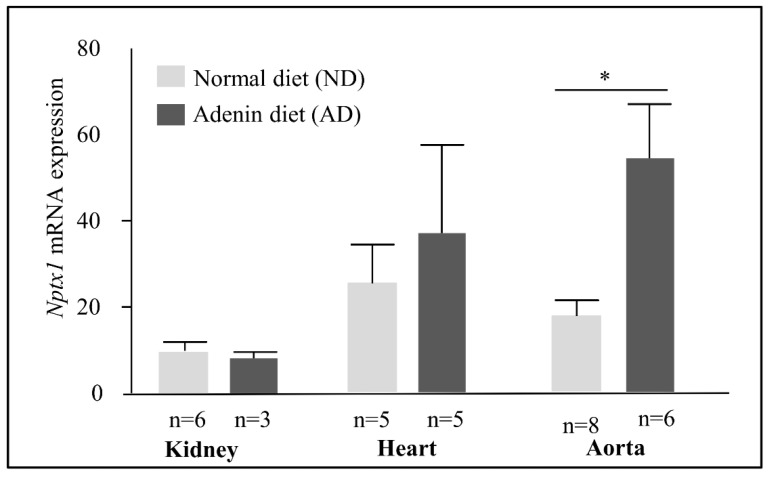
Effects of CKD of Nptx1 mRNA levels. Nptx1 mRNA levels in heart and Aorte of mice with normal diet (ND) or adenin diet (AD). Values are expressed as mean ± SD. Mann Whitney test was used and a *p* value < 0.05 was considered significant (*).

We used mice fed a high-adenine diet as a model of CKD [28,29]. In excess, adenine is metabolized by xanthine dehydrogenase (XDH) into 2,8-dihydroxyadenine (DHA), which precipitates in the tubules of kidneys and thus leads to interstitial tubulointerstitial nephropathy. The expression of *Npx1* in the kidneys, heart and aorta was low. In mice with CKD, we observed no increase in NPTX1 mRNA levels in the heart and in the kidneys but an increase in the aorta (Figure 6).

## 3. Material and Methods

### 3.1. Cell Culture and Treatments

Human umbilical vein endothelial cells (HUVEC) are isolated from the veins of the umbilical cord by collagenase digestion, as previously described [30]. Briefly, the cord severed from the placenta after birth was infused with collagenase. After incubation, the endothelial cells were flushed from the cord by perfusion with buffer, grown in EGM-2 medium, and used until the fifth passage maximum. IAA, IS, 2, 3, 7, 8-tetrachlorodibenzo-p-dioxin (TCDD), DMSO and FICZ were purchased from Sigma (Saint Quentin Fallavier, France). Since IS was a potassium salt, we used KCl at an equivalent concentration as a control. (Sigma, Saint Quentin Fallavier, France). Uremic solutes TCDD and FICZ were compared with their respective controls (ethanol for IAA; KCl for IS; DMSO for TCDD and FICZ) diluted in the culture medium in the same concentration.

### 3.2. Mice 

C57BL/6J wild-type mice were purchased from The Jackson Laboratory and maintained as a breeding colony in the animal care facility at the Faculty of Medicine of Marseille. When they reached 13 weeks of age, the male mice were fed ad libitum with a 0.25% adenine-enriched diet (A04 + 0.25% adenine, SAFE, Augy, France) for 2 weeks to induce uremia. Then, adenine was withdrawn, and mice were fed with the regular chow diet for another week, as previously described [28]. The mice of the control group were only fed a regular chow diet (A04 standard, SAFE).

### 3.3. Total RNA Extraction from HUVEC and Mouse Organs 

Total RNA was extracted from HUVEC by an RNeasy mini-kit (Qiagen, Courtaboeuf, France). Mouse organs were lysed in TRIzol and total RNA was purified by chloroform extraction and isopropanol precipitation. RNA concentration was determined using a NanoDrop^®^ Spectrophotometer (Thermoscientific, Wilmington, DE, USA).

### 3.4. Comparative Quantification of mRNA Levels 

Gene expression was analyzed by reverse transcription and comparative polymerase chain reaction (RT-PCR). Reverse transcription using random primers and oligodT was performed on 500 ng of total RNA using the Takara PrimeScript RT Reagent Kit (Ozyme, Saint Quentin en Yvelines, France), followed by PCR on 12.5 ng of cDNA using the Takara SYBR qPCR Premix Ex Taq (Ozyme, Saint Quentin en Yvelines, France) for human primers and the taqman system (the Brilliant II QPCR Master Mix (Agilent Technologies, Les Ulis, France) for mouse primers. PCR reactions were made with OneStepPlus (Thermofisher, Courtaboeuf, France) using comparative quantification of mRNA levels. The results were normalized with the housekeeping gene *HPRT* (for HUVEC) or *Gus* (for mice). The fusion curves were analyzed to assess the specificity of the detected fluorescence. The sequences of the primers for *HPRT, AHR* and *NPTX1* are listed below:

*HPRT* (*HPRT1*;HGNC:5157)
HPRT-F 5′GGATTATACTGCCTGACCAAGGAAAGC3′
HPRT-R 5′GAGCTATTGTAATGACCAGTCAACAGG3′
*AHR* (*AHR*;HGNC:348)
AHR-F 5′TGTTGGACGTCAGCAAGTTC3′
AHR-R 5′TGGTGCCCAGAATAATGTGA3′
*NPTX1* (*NPTX1:HGNC:7952)*
NPTX1-F 5′TGTTGGACGTCAGCAAGTTC3′
NPTX1-R 5′TGGTGCCCAGAATAATGTGA3′

Primers and MGB-Taqman probes were purchased from Thermofisher (*nptx1* and the housekeeping gene *Gusb*). Target gene expression in mice was normalized on the basis of the *Gusb* content of each sample and was subsequently normalized to a basal mRNA level with the equation: N target = 2 ΔCt sample, where ΔCt is the Ct value of the target gene minus the Ct value of the *Gusb* gene. The results are reported as normalized mRNA levels, i.e., the N target value divided by the N target value of the smallest quantifiable amount of target gene mRNA (target gene Ct value = 35).

### 3.5. Gene Silencing

*AHR* mRNA levels were decreased using a pool containing three siRNAs directed against AhR (1200, 1999 and 1998, 10 nM each, Thermofisher, Courtaboeuf, France) and the siPORT™ Amine Transfection Agent (Thermofisher, Courtaboeuf, France) according to the manufacturer’s instructions. siRNA control (SignalSilence^®^ Control siRNA Unconjugated, Ozyme, St Quentin en Yvelines, France) was used at a concentration of 30 nM. Briefly, cells were seeded 24 h before transfection, at a density of 2 × 10^5^ cells by wells in 6 Well Culture Plate precoated with gelatine 0.2% (Thermofisher, Courtaboeul, France). The three siRNAs diluted in 100 µL of OPTI MEM medium were gently mixed with 100 µL of OPTI MEM containing transfection reagent and incubated for 10 min at room temperature to allow complex formation. Just before transfection, the culture medium was removed and 2 mL of OPTI MEM were added to each well. A 200 µL de mixture containing siRNA and transfection reagent was added to the cell. The culture medium was changed the next day. Since we have previously shown that the decrease in AhR mRNA level was achieved 24 h after transfection and remained stable until 96 h [24], the effect of IS and/or IAA on mRNA and protein were investigated 48 h after transfection.

### 3.6. Protein Extraction from HUVEC and Western Blot

After cell incubation with AhR agonists or controls, cell culture flasks were placed in ice and washed with cold PBS. Then cold RIPA buffer (50 mM Tris-HCl, 150 mM NaCl, 0.1% sodium dodecyl sulfate (SDS), 1% Triton X-100, 1 mM EDTA, pH 7.4) containing protease inhibitors (Complete mini, Roche Diagnostics France, Meylan, France) and proteins were extracted as previously described [29]. Protein concentrations were measured with the Bicinchoninic Acid Kit for Protein Determination (BCA1, Sigma Aldrich, St. Quentin Fallavier, France). Equal amounts of total protein (40 μg) from cell lysates were loaded on 4–12% sodium dodecyl sulfate–polyacrylamide electrophoretic gel and transferred onto a polyvinylidenedifluoride membrane. Nonspecific binding was blocked by immersing the membrane in PBS-5% BSA at room temperature for 1 h. The membrane was incubated with a primary antibody directed against NPTX1 (Becton Dickinson, Le Pont de Claix, France) and actin, the loading control (1:1000 dilution; D6A8, Cell Signalling, Yvelines, France). The secondary peroxidise conjugated antibody, used at a 1:2000 dilution, was purchased from Beckman Coulter. Revelation was made by chemiluminescence (ECL Western blotting substrate, Pierce, Courtaboeuf, France) and the gel image was captured using the Syngene GBox (Ozyme, Saint Quentin en Yvelines, France). Densitometry analyses of chemiluminescence staining were performed with the software *ImageJ* (Rasband, W.S, Bethesda, ML, USA). Results were expressed as a ratio between values obtained with AhR agonists and the value obtained with the control, and then normalized with values obtained with actin. 

### 3.7. Statistical Analysis

Statistical analysis was performed with Prism software (GraphPad Software Inc., San Diego, CA, USA). Wilcoxon matched-pairs signed rank test and Mann–Whitney test were used. Differences were considered significant when *p* was less than 0.05. 

## 4. Discussion

In this work, we showed that two uremic indolic solutes induced the expression of NPTX1 in primary endothelial cells in an AhR-dependent way. In the adenine mouse model of CKD, increased levels of *Nptx1* in the aorta were also observed, suggesting that there may be an upregulation of *Nptx1* during renal disease. This effect was not agonist-dependent, since two other AhR agonists, FICZ and TCDD, belonging to structurally distinct compounds also increase NPTX1 expression.

AhR is a transcription factor involved in detoxification [31,32], development [33,34], metabolism [35,36], immunity [37,38,39] and cardiovascular homeostasis [14,40]. The detoxification pathway, first identified in patients contaminated by the dioxin TCDD, is illustrated by the signature of AhR activation, which displays upregulation of genes involved in transformation of molecules until their extrusion from the cell [32]. This function is no doubt also related to the others, since transformation of endogen AhR agonists by enzymes alters the metabolome composition and consequently the cellular signaling pathways. In addition to this canonical signature pattern, AhR activation modifies the expression of numerous other genes in a cell- and agonist-specific way, explaining the diversity of AhR effects [32]. Our previous experiments on arrays allowed us to show for the first time that tissue factor, the trigger for coagulation, was also a target of AhR activation by IS and IAA [24]. From these results obtained in vitro, we and other researchers also demonstrated that patients and animals with CKD display both AhR pathway activation and increased tissue factor expression and activity [23,24,28,41,42]. AhR activation and increased expression of one of its targets, tissue factor, have particular implications in light of the increased risk of thrombotic events in CKD [43,44]. Thus, tissue factor upregulation by IS and IAA could be a mechanism behind high cardiovascular mortality and especially the increased thrombotic risk in CKD. 

Here, we showed that NPTX1 is also a target of indolic uremic toxins. Furthermore, *Nptx1* is upregulated in the aorta of uremic mice, suggesting that the induction by IS and IAA observed in vitro in endothelial cells may occur in vivo during kidney failure. Chitalia et al. established a useful and relevant animal model to study AhR activation in organs during CKD [45]. A transgenic mouse line expressing the AHR responsive promoter tethered to a β-galactosidase reporter gene was fed with adenine to develop CKD [45]. β-galactosidase expression in these mice reflects AhR activation and was observed in cardiomyocytes and in the vascular wall of the aorta, including endothelial cells. Moreover, the fact that in these mice, AhR activation was associated with the IS serum level indicates that IS plays a role in AhR activation in CKD mice. Since we performed RT-PCR experiments on aorta homogenates, we were unable to link NPTX1 increased expression to a specific cell type. However, the results obtained in transgenic mice by Chitalia et al. make it plausible that endothelial cells were able to express NPTX1. In contrast, no increase in *Nptx1* expression was observed in the heart and kidneys of CKD mice. These results suggest that NPTX1 was not involved in injuries or homeostasis during CKD in these organs. 

NPTX1 belongs to the conserved pentraxin family protein, which is characterized by the presence of the 200 amino-acid-long pentraxin domain [25,46]. The short pentraxin group consists of the C reactive protein (CRP) and the serum amyloid protein (SAP). The long pentraxin group consists of pentraxins 1 to 4 and the pentraxin receptor (NPR), and has supplementary domains in the C-terminal part [46,47]. CRP, SAP and pentraxin 3 are inflammatory proteins linked to innate immunity [46,47,48]. NPTX1 has a broader range of functions, from a structuring role in synapse genesis to a proapoptotic role under stress. In neurons, where it was first identified, NPTX1 plays a role in synapse formation by regulating the expression and positioning of glutamate receptor 1 and potassium channels via a complex network of signals and interactions involving NP2, NPR and glypican 4 [49,50]. NPTX1 plays a further role in neurons under conditions of stress, such as hypoxia and potassium deprivation, by triggering apoptosis in a bax3-dependent fashion [51,52,53,54]. Later, NPTX1 expression was no longer found to be restricted to the nervous system and was also observed in pancreatic cells [55], liver cells [56], olfactive mucosal cells [57], colon cells [58], myogenic stem cells [59] and endothelial cells [60]. In these cells, non-a priori experiments, such as microarrays or subtractive libraries, revealed that this protein is a consequence of cell exposure to deleterious situations, for example to a high glucose level [55], or to particle pollution in the case of olfactive mucosal cells [57]. The role of this inducible NPTX1 is pleiotropic; in most cells, NPTX1 induces apoptosis, as it does in neurons, whereas in olfactive mucosal cells, NPTX1 no longer acts as an apoptotic trigger but actually protects these cells against oxidative stress and mitochondria dysfunction induced by particulate matter [57]. Indeed, canceling NPTX1 expression by silencing RNA amplifies the oxidative stress induced by particles. 

IS and IAA have been shown to induce oxidative stress in endothelial cells even when these cells are subjected to protective shear stress [10,22]. FICZ also induces oxidative stress in keratinocytes [61,62] and TCDD induces mitochondrial dysfunction by generating ROS in hepatocytes [63]. In our experiments, at the concentration used, IS and IAA do not induce cell mortality [22,24]. Thus, it could reasonably be hypothesized that NPTX1 induction by these agonists serves to prevent ROS generation, as observed in olfactive mucosal cells, and not to induce apoptosis. To test this hypothesis, it would be interesting to prevent the induction of NPTX1 by silencing RNA and then to evaluate the level of oxidative stress induced by these AhR agonists. If the stress decreased, this would confirm that NPTX1 plays a protective role, preventing strong oxidative stress and the associated damage, being part of the detoxification process orchestrated by AhR. On the other hand, if NPTX1 continuously increased in vivo during the progression of renal disease, as observed in the aorta of CKD mice, this might indicate that the impact of NPTX1 can be deleterious. Indeed, Eckers et al. demonstrated that activation of AhR in the vasculature interferes with endothelial nitric oxide synthase activation and found a positive correlation between AhR expression and vessel stiffness in healthy human subjects [40].

The four AhR agonists we used display chemically different structures. IS and IAA are indolic compounds, with a sulfate group for IS and an acid acetic group for IAA. FICZ also contains an indole, but with a very large carbazole group [64]. IS, IAA and FICZ are endogenous compounds produced from tryptophan but by different pathways and body parts. IS originates from microbiota [8,14,65], IAA from microbiota and tissues [14,66], and FICZ from skin [20]. TCDD, an environmental pollutant that led to the discovery of AhR, is a polychlorinated dibenzo-p-dioxin [67]. This diversity in structure is illustrated by different affinities for AhR [31,32,64]. However, all these agonists can induce NPTX1 expression, suggesting that NPTX1 is an AhR target that is not agonist-specific. The fact that inducible NPTX1 expression is observed in different cell types under conditions of stress also suggests that it is not cell-specific. Thus, NPTX1 could belong to the signature of AhR activation. Finally, these first results showing NPTX1 as a new target of AhR allow us to identify 3 full-length consensus sequences for AhR binding in the first 1500 nucleotides of the NPTX1 promoter and 4 shorter consensus sequences. 

All concentrations used for IS (2 to 500 µM) increased endothelial NPTX1 mRNA levels to the same extent. For IAA, the effect on the NPTX1 mRNA level was obtained only with the higher concentrations (25 to 75 µM). This could be explained by the presence of two different chemical groups linked to the indole ring that may affect interactions with AhR and consequently its activation. In addition, almost all the concentrations of uremic toxins used for these in vitro experiments can be found in the serum of patients with CKD: 2 to 200 µM for IS and 2 to 25 µM for IAA [21,23,24]. Therefore, these two toxins could exert their toxicity in vivo. However, we do not know whether there is synergism or competition between them; if they exhibit structural similarity with the indole group, the sulfate and acetic groups could induce specific interactions in the AhR binding pocket. In patients with an IAA concentration of below 25 µM, IS alone could be responsible for an increase in NPTX1; however, we cannot rule out the possibility that IAA heightens the action of IS. Further biochemical studies would help explain the molecular mechanisms of the interactions between these two indolic toxins and AhR.

In conclusion, we demonstrated for the first time here that two indolic uremic solutes increased a new AhR target, NPTX1. This calls for further exploration to fully elucidate the role of NPTX1 both in the AhR pathway and in renal disease. This finding offers new hope of improving our knowledge of the complex and interrelated mechanisms of complications associated with CKD. The ultimate goal should be to identify new effective treatments, since the many conventional therapeutic strategies for CVD have been unsuccessful in providing a survival benefit for patients with CKD [68].

## Data Availability

All data are available.
